# Healthcare Practitioners' Knowledge of Lymphedema

**DOI:** 10.1155/2021/3806150

**Published:** 2021-12-31

**Authors:** Hossein Yarmohammadi, Amirhossein Rooddehghan, Masood Soltanipur, Amirabbas Sarafraz, Seyed Fatah Mahdavi Anari

**Affiliations:** ^1^Medical Students Research Committee, Shahed University, Tehran, Iran; ^2^Department of Laboratory Sciences, Faculty of Paramedical Sciences, Tehran Medical Sciences, Islamic Azad University, Tehran, Iran; ^3^Pavlov First Saint Petersburg State Medical University, Saint Petersburg, Russia; ^4^Department of Dermatology, Shiraz University of Medical Sciences, Shiraz, Iran

## Abstract

**Objectives:**

Lymphedema is neglected in medical education, and a review on healthcare practitioners' (HCPs) knowledge is necessary to shed light on gaps and to provide evidence for establishing educational programs on lymphedema.

**Methods:**

This systematic review was performed based on the PRISMA guideline in PubMed, Scopus, Web of Science, and Google Scholar databases. There was no limitation on the type of lymphedema or HCPs. The quality assessment was performed based on QATSDD. Data regarding study characteristics, questionnaire context, and findings of the study were summarized from each article.

**Results:**

After the screening, 16 articles were included that 12 were cross-sectional, two were qualitative, and two were interventional pilot studies. Breast cancer and other cancer-related lymphedema, lymphatic filariasis, and podoconiosis were included, and the majority of articles were focused on primary HCPs. The overall knowledge was low and average in five and 11 articles, respectively, and prior education was a significant factor related to higher knowledge of lymphedema in two studies.

**Conclusion:**

Structured education of lymphedema is needed to increase the knowledge of HCPs and to enhance their collaboration in multidisciplinary care teams. Improvement of HCPs' knowledge may lead to better outcomes of lymphedema patients' management which are neglected.

## 1. Introduction

Lymphedema is the malfunction of lymphatic circulation that leads to interstitial fluid accumulation in tissues. This condition affects quality of life (QoL) and increases the risk of complications such as cellulitis, obesity, skin changes, and susceptibility to cancer [1–4]. Lymphedema is mainly categorized into two major types: primary lymphedema which results due to genetic developmental abnormalities resulting in lymphatic vasculature malformation or malfunction and secondary lymphedema that is usually acquired after an injury to lymphatic vessels [5]. In developed countries and many other regions, secondary lymphedema is mainly considered as phlebolymphedema which arises in the context of chronic venous insufficiency (CVI). The other cause is due to a complication of cancer treatment, specifically after radiotherapy or surgery for breast cancer which puts survivors at risk for breast cancer-related lymphedema (BCRL) [6–8]. While in some other regions dealing with neglected tropical diseases (NTDs), the more important form of secondary lymphedema is lymphatic filariasis or podoconiosis [9, 10]. Nevertheless, despite the great impact of lymphedema on healthcare systems worldwide, it has been neglected in medical research and education [11].

Lymphedema is a chronic disease with no cure, and therefore, patients' adherence to currently standard management including complete decongestive therapy (CDT) is essential [2]. The gap in lymphedema management to some extent may be attributed to patients' lack of knowledge toward routine self-care and prevention [12]. Patients' education has been shown to decrease the incidence and severity of lymphedema among cancer survivors [13, 14], and also, community education regarding the nature of filariasis and podoconiosis has led to reduced stigmatized behaviors [15]. The education of patients or at-risk populations is dependent on the knowledge of healthcare providers about lymphedema [16]. The engagement of different practitioners such as primary care physicians, nurses, physiotherapists, surgeons, oncologists, dermatologists, psychiatrists, and other specialties involved in lymphedema management shows the diverse nature of lymphedema education for healthcare providers [2].

Since lymphedema is not addressed adequately in medical research and education and it already may have been in the wayside of healthcare delivery in different countries [17, 18], it is important to find the gaps in healthcare practitioners' (HCPs) knowledge that could be an opportunity for proper interventions.

## 2. Methods

### 2.1. Search Strategy

The electronic search was conducted in PubMed, Scopus, and Web of Science and updated on 20.11.2021 for the last time. Also, the references of included articles were screened for relevant studies, and an extra manual search was done in Google Scholar to cover possible additional related articles. The searched strategy was the combination of keywords including “lymphedema”, “lymphoedema”, “filariasis”, “podoconiosis”, “phlebolymphedema” and “knowledge”. This study was performed based on the Preferred Reporting Items for Systematic Reviews and Meta-Analyses (PRISMA) guideline [19].

### 2.2. Eligibility Criteria

Articles that had investigated the knowledge of HCPs in any field regarding any type of lymphedema were included. HCPs were considered but not limited to nurse, physician, pharmacist, physiotherapist, etc., practicing as a primary care provider or in any specialty such as oncology, surgery, and others. Lymphedema types included primary lymphedema, cancer-related lymphedema, lymphatic filariasis, podoconiosis, phlebolymphedema, or any other type. The anatomical site of lymphedema was no exclusion criteria, and lymphedema at extremities, genital, head, and neck or any other site were eligible.

Studies on the knowledge of other populations than HCPs such as patients were excluded. Since it was important to demonstrate specific gaps in the knowledge of lymphedema in this systematic review, articles that had not reported adequate information were excluded; for example, studies that had investigated the knowledge of HCPs regarding cancer survivorship which the reported result related to lymphedema was not enough to show the knowledge gap were not included. As well, studies that had reported knowledge regarding filariasis management which mainly were focused on parasitological concepts rather than lymphedema itself were excluded.

There was no restriction on the year of publication; however, only articles in English were included. There was no limitation on the design of original studies, and both qualitative and quantitative were eligible, although interventional studies were not included unless they had provided information on the knowledge before the intervention. Therefore, reports of newly established academic programs for lymphedema education among HCPs with no information on education level were excluded. Abstracts, letters, commentaries, books, and reviews were not eligible, but references were screened for relevant articles. Additionally, non-peer-reviewed manuscripts and theses were excluded.

The screening of search results was done by two authors independently, and the third author was asked to decide in the case of disagreement. Titles and abstracts were screened, and then, relevant full texts were determined for data extraction.

### 2.3. Data Extraction and Synthesis

Included articles were reviewed by two authors independently, and the following characteristic data were extracted of each article: the first author, year of publication, the country, the type of lymphedema, population, and study design. In the case of disagreement, the third author was invited to decide.

The type of questionnaire for each article was determined. Questions in the questionnaire of each study were gathered and categorized into 5 main key concepts related to lymphedema: (A) Lymphatic System, (B) Prevention, (C) Diagnosis, (D) Management, and (E) Complications.

The reported knowledge in included articles was extracted. The overall knowledge in each study was determined as low, averaged, or high. This judgment was made by two authors independently based on the results of the study. Additionally, any other reported results of knowledge in numbers, percent, comparisons, etc., were extracted. If in the article, any factor related to the lymphedema knowledge was provided; it was gathered, and those factors with statistical significance were marked.

Gaps in knowledge were primarily considered as those questions and key concepts with lower knowledge; however, any specifically mentioned gap at the included article was extracted. Other findings, rather than knowledge and its related factors, were collected as secondary outcomes such as attitude and practice of HCPs. And finally, if any suggestions had been made in included articles were determined, such as proper interventions to improve knowledge and the appropriate type of lymphedema education for HCPs.

All extracted data were summarized and tabulated and were confirmed by the third author at last.

### 2.4. Quality Assessment

Quality Assessment Tool for Studies with Diverse Designs (QATSDD) was used for quality assessment [20]. This tool consisted of 16 questions that could be used for both qualitative and quantitative studies. Since each question had a possible score from 0 to 3, the overall possible score was 42. Two authors independently judged articles, and a senior author was asked for the final judgment in the case of discrepancy. The final score for each article was calculated as a percent out of 42, and the range of scores for all articles was determined to show the overall quality of included articles.

## 3. Results

**Figure 1:**
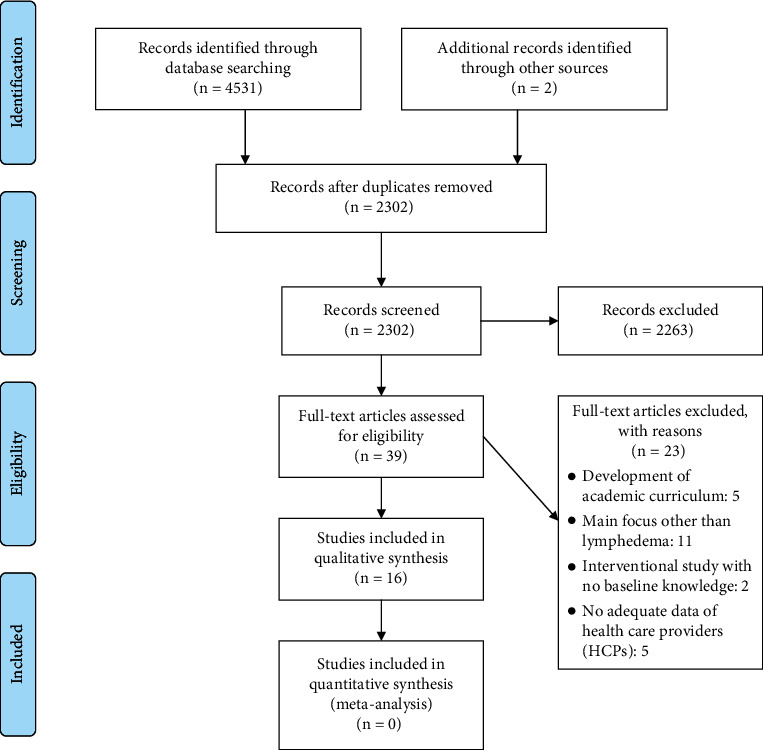
The PRISMA flowchart of included studies on knowledge of HCPs of lymphedema.

After screening, 16 articles met the inclusion criteria [21–36].  Figure 1 shows the PRISMA flow chart of included and excluded articles.

### 3.1. Lymphedema and Study Design

Although in five articles, the type of lymphedema was not determined, and it was generally addressed to any type of lymphedema [21, 22, 25, 34, 36]; different types were studied, including BCRL [27, 29, 30], cancer-related lymphedema [28, 31, 33], filariasis [23, 35], and podoconiosis [23, 24, 26, 32]. Interestingly, one article was focused only on genital lymphedema [21]. The design of studies was cross-sectional and qualitative in 12 [21, 22, 24–32, 36] and two [34, 35] articles, respectively, while two studies were interventional [23, 33]. These characteristics are provided in Table 1.

### 3.2. Population

The number of the studied population varied between 18 and 867. Five studies had assessed knowledge in nurses only [25, 27, 28, 31, 34] while other articles had studied oncologists, radiologists, plastic surgeons, oncology surgeons, general physicians, family medicine residents/specialists, physiotherapists, occupational therapists, environmental officers, laboratory and pharmacy technicians, and others. Primary care and community care practitioners were the main target population in almost all of the included articles. Further information is provided in Table 1.

### 3.3. Questionnaire and Key Concepts

The knowledge assessment in seven articles was not based on measurable results of a questionnaire and was self-reported or interviewed [21, 22, 25, 28, 33–35]. Despite that, the key concept of the questionnaire in two articles was not provided [22, 29]; each key concept of prevention (B) and management (D) was addressed in 13 articles. The lymphatic system (A), diagnosis (C), and complications (E) were present in the questionnaire of 11, 12, and six articles, respectively. The most frequently question in questionnaires was mainly focused on etiology (A) [23, 24, 26, 31–34], risk factors (B) [21, 24, 27, 28, 30, 31, 33, 34, 36], signs and symptoms (C) [24–26, 28, 30–33], being curable (D) [23, 25, 26, 30, 33, 35, 36], and psychosocial impact (E) [21, 33, 36]. The questionnaire type and key concepts are summarized in Table 2.

### 3.4. Knowledge and Related Factors

The overall knowledge was low and averaged in five [26, 27, 34–36] and 11 [21–25, 28–33] articles, respectively. Different factors were studied in nine articles for their possible relationship with HCP knowledge [21, 24, 26, 27, 29–32, 36]. Academic qualification, profession, and experience were more frequently reported among factors that showed a significant relationship with knowledge. Previous education on lymphedema was reported in four articles that in two were related to knowledge significantly [29, 30]. More details on knowledge and its related factors are presented in Table 2.

### 3.5. Gaps, Other Relevant Findings, and Suggestions

Etiology, signs and symptoms, and management such as skincare were among frequently mentioned gaps in knowledge. The other relevant findings were reported such as attitudes, gaps in practice, and referral patterns. Also, eagerness for further lymphedema education was evident in the majority of the study population in four articles [23, 27, 28, 36]. Different types of education delivery were suggested in almost all of the included articles which are provided in Table 3.

### 3.6. Quality Assessment

The lowest and highest scores were 24 (57.1%) [36] and 40 (95.2%) [24, 26, 30], respectively. Therefore, all included articles were scored at least the half of possible score or more based on QATSDD [20]. The score for each study is presented in Table 1.

## 4. Discussion

In this systematic review, 16 articles were included, and the knowledge was low to average. Limited studies support the significant relationship between knowledge and previous education, and additionally, an educational intervention was suggested in almost all of the included articles. Apparently, there has been an increased interest in this topic since half of the included articles were published during the previous four years.

### 4.1. Neglecting Lymphedema

Lymphedema includes a wide range of different types with diverse geographical distribution patterns globally. In this systematic review, articles from different countries were included. The study of Schulze et al. in 2018 reported heterogeneity of lymphedema professionals worldwide [18]; however, it concluded a gap in lymphedema care and suggested structured education and knowledge certification of lymphedema.

Unfortunately, there was no report on knowledge of phlebolymphedema. This condition is related to CVI, and although limited articles have been published on this topic compared to cancer-related lymphedema, some experts in recent articles recognize it as the main cause of secondary lymphedema in Western countries [8, 37]. The knowledge of phlebolymphedema seems to be an important research question for HCPs, specifically cardiologists, radiologists, and vascular surgeons.

Interestingly, a recent article investigated the knowledge of genital lymphedema among HCPs [21]; however, head and neck lymphedema was not studied in any article. These lymphedemas are mainly caused due to cancer treatment or filariasis [38, 39]. Compression therapy is more difficult in these regions, and surgical options include different interventions based on nonphysiologic or physiologic procedures [40, 41]. Although lymphedemas which affect other regions than upper and lower limbs may require a higher level of expertise, the absence of such topics in literature is not favorable. Any structured lymphedema education is better to at least mention key points of genital and head and neck lymphedema management [40].

### 4.2. Knowledge of Lymphedema

Most of the questionnaires were focused on prevention (B) and management (D) of lymphedema. The standard management of lymphedema is CDT consisting of manual lymphatic drainage (MLD), compression therapy, exercise, and skincare [2]. However, CDT has limitations in higher stages of lymphedema and difficulties with patients' adherence to therapy. There has been an interest in surgical intervention for lymphedema prevention or treatment during recent years [42]. Surgical interventions for lymphedema include nonphysiologic (such as surgical excision or liposuction) and physiologic (such as vascularized lymph node transfer or lymphovenous anastomosis) procedures. Also, other surgical approaches to prevent cancer-related lymphedema now are available such as the lymphatic microsurgical preventive healing approach. It seems that the management of lymphedema will be more dependent on a combination of both conservative and surgical therapies, which means the necessity of a trained multidisciplinary team of different specialties and expertise [43].

Other modalities for lymphedema management also have been investigated such as laser therapy or pneumatic compression pumps [44–46], but they have not been based on recent findings of lymphedema pathophysiology [47, 48]. Only one included study in this systematic review had a question of pathophysiology (A) in the questionnaire [27], and this indicates the importance of more emphasis on the recent untranslated findings both in research and practice of lymphedema.

Early diagnosis and referrals were addressed as prevention (B) or diagnosis (C) questions. Literature supports early detection of lymphedema since it can prevent further advanced stages and fibroadipose changes in tissue. Early referrals are impossible without alert HCPs with substantial knowledge of lymphedema [49] which again highlights the importance of a multidisciplinary approach to lymphedema management [50, 51].

The key concept of lymphedema complications (E) was addressed less than other concepts which indicate the possibility of neglecting complications such as psychosocial impact. Although the psychosocial impacts of lymphedema such as body image and QoL disturbances and depression have been acknowledged widely in the literature, evidence on the economic burden of lymphedema is limited [52]. Increasing awareness of such burden may change HCPs' attitude toward lymphedema management and decrease its neglect in research and education.

Additionally, favorable attitudes (with no stigmatization) toward podoconiosis and lymphatic filariasis had a relationship with knowledge in two included studies [24, 32]. The psychosocial factors in lymphedema management have a significant role in developing countries. The study of Kouassi et al. in the Republic of Guinea in 2018 showed the strong impact of religious beliefs on lymphedema management [53]. Future studies need to pay more attention to such socio-cultural factors and suggest feasible delivery of education in this context.

Also, cellulitis as a well-known complication of lymphedema was only addressed in the questionnaire of one study, while its diagnosis is an obstacle for HCPs and it seems more research in this field is needed [54]. Cellulitis is considered a prevalent complication as one in three individuals affected with lymphedema experience at least an episode, and it increases in higher stages of lower extremity lymphedema [8]. Skin changes, susceptibility to malignancy, and obesity are other recognized complications [1, 3, 4]; however, there has been recent interest in musculoskeletal complaints, bone density changes, and apnea as complications of lymphedema which also negatively affect QoL [55–57], and none were addressed in questionnaire of any included article.

### 4.3. Education of Lymphedema

The major focus of included articles was on primary care providers, and the knowledge of these practitioners was reported to be less compared to professionals [58]. Although the healthcare system is diverse among different countries, primary care practitioners play an important role in cancer survivorship and also in community programs of NTD control. Some medical procedures have been suggested as risk factors of lymphedema such as injection and blood pressure measurement, and traditionally, HCPs are suggested to avoid such procedures [59]. Therefore, HCPs need to be aware of lymphedema preventive measures. Also, their awareness of early lymphedema diagnosis decreases costs and complications such as cellulitis [60]. One of the main gaps of knowledge was the timing and setting of referral to lymphedema specialists in included articles and also somehow confusion of HCPs (primary care nurses for example) of their role in lymphedema management. This finding highpoints the importance of an inclusive perspective of lymphedema knowledge, not only for primary care physicians and nurses or surgeons and oncologists but also for other specialties such as orthopedic surgeons, dermatologists, plastic surgeons, etc. Some lymphedema patients may need a referral to other specialties for complications such as carpal tunnel syndrome and other musculoskeletal disorders, their skin/wound care, and surgical intervention for those patients unresponsive to CDT. Therefore, one of the objectives of lymphedema education for HCPs should be focused on increasing practitioners' capability of proper referral and communication with other specialties in a multidisciplinary approach for improving outcomes of lymphedema management [50, 51]. Different suggestions were made in included articles, and some had considered undergraduate training of lymphedema, and yet, no study had investigated the knowledge of undergraduate students' knowledge of lymphedema as the main target population. Literature supports the neglect in the education of lymphology and lymphatic system, and lymphedema could be a reasonable part of expanded lymphology education in the future [61, 62].

### 4.4. Limitations

The electronic search had limitations, and only articles in English were included. Therefore, there may be some other evidence that has not been included in this systematic review. Also, since the aim of this study was to focus on lymphedema, other articles reporting knowledge of HCPs of cancer survivorship and NTD control programs were excluded, and there has been extensive research in such fields in the literature while lymphedema has not been addressed properly.

## 5. Conclusion

The HCP's knowledge of lymphedema is not favorable based on reports in the literature, and more emphasis should be made on current gaps in the field. Also, efforts for establishing a multidisciplinary approach for lymphedema research, education, management, and policymaking should be taken. Increasing knowledge of HCPs may probably lead to better outcomes of patients' management, and structured education programs need to emphasize more on the referral patterns of patients in a multidisciplinary care team to enhance the care delivery of neglected individuals with lymphedema.

## Figures and Tables

**Table 1:** Characteristics and quality of included articles.

Author, year	Country	Lymphedema type	Population: number (specialities or practice settings) = total number	Study design	Study quality (%)
Noble-Jones et al., 2021 [21]	UK	NA	Nurses (46%), physiotherapists (21%), occupational therapists (3%), uro-oncology nurse, urology surgeon, pelvic specialist physiotherapist and radiologists (30%) = 149	Cross-sectional	37 (88.0)
Omar et al., 2021 [22]	Saudi Arabia	NA	Physical therapists (18), occupational therapist (1) = 18	Cross-sectional	27 (64.2)
Dellar et al., 2021 [23]	Ethiopia	Filariasis, podoconiosis, leprosy	Nurses (49), midwives (9), health officers (23), pharmacists (9), laboratory technicians (9) = 35	Interventional education	39 (92.8)
Churko et al., 2021 [24]	Ethiopia	Podoconiosis	Nurse: 220, midwives: 16, health officer: 57, laboratory technician: 18, pharmacy: 9 = 320	Cross-sectional	40 (95.2)
Pereira Rios Gerez et al., 2020 [25]	Brazil	NA	WOS nurses = 97	Cross-sectional	27 (64.2)
Bayisenge et al., 2020 [26]	Rwanda	Podoconiosis	Physicians: 13, nurses/midwives: 59, CHP: 226, environmental officers: 38 = 336	Cross-sectional	40 (95.2)
Abu Sharour, 2019 [27]	Jordan	BCRL	Oncology nurses: 150 (surgical wards and out-patient clinics)	Cross-sectional	36 (85.7)
Tsuchiya et al., 2018 [28]	Japan	Cancer-related	Public health nurses: 641 (government employed)	Cross-sectional	34 (80.9)
Mete Civelek et al., 2015 [29]	Turkey	BCRL	Family physician: 116, family medicine resident: 68, family medicine specialist: 130 = 314	Cross-sectional	36 (85.7)
Tam et al., 2012 [30]	USA	BCRL	Surgeon: 85, oncologist: 50, primary care physician: 658, primary care nurse: 74 = 867 (general, breast and plastic surgery, medical and radiation oncology, family medicine, internal medicine, obstetrics/gynecology)	Cross-sectional	40 (95.2)
Ryan et al., 2012 [31]	USA	Cancer-related	Oncology advanced nurses: 238 (medical oncology, blood/marrow transplantation, palliative care, prevention/detection, radiation oncology, surgical oncology, others)	Cross-sectional	39 (92.8)
Yakob et al., 2009 [32]	Ethiopia	Podoconiosis	Nurse: 186, health assistant: 42, laboratory technician: 19, pharmacy technician: 18, physician and health officers: 7 = 272	Cross-sectional	37 (88.0)
Mathews et al., 2007 [33]	Canada	Cancer-related	Healthcare professionals: 80 (nurses, nursery students, others)	Interventional education	33 (78.5)
Morgan et al., 2005 [34]	UK	NA	Community nurses: 54	Qualitative	39 (92.8)
Rath et al., 2005 [35]	India	Filariasis	Peripheral primary care: 41	Qualitative	27 (64.2)
Logan et al., 1996 [36]	UK	NA	Peripheral primary healthcare: 339 (general practice, practice nurses, physiotherapists)	Cross-sectional	24 (57.1)

%: percent of maximum possible quality (42); UK: United Kingdom; NA: not available; CHP: community health practitioner; BCRL: breast cancer-related lymphedema; NGO: nongovernmental organization; USA: United States of America; WOS: Wound, ostomy, and incontinence.

**Table 2:** Key concepts of questionnaire, knowledge results, and its related factors.

Author	Assessment tool	Knowledge key concepts^⸸^	Knowledge results	Knowledge related factors
Noble-Jones et al. [21]	Self-reported	A: male and female anatomy B: surgical risk factors C: self-report tools, assessment and evaluation, clinical reasoning D: MLD and SLD, skin care, bandaging and taping, compression and pneumatic pumps, electrotherapy, exercise, factors affecting therapy E: legal, cultural, and ethical considerations	Averaged knowledge (i) 1.88 out of 3 (without genital lymphedema education: 1.74, with education: 2.02) (ii) Self-reported knowledge on treatment techniques > theoretical background	Experience^∗^, previous education

Omar et al., 2021 [22]	Self-reported	NA	Averaged knowledge (i) Excellent or very good: 83%, good, average or normal: 17% (ii) Need for further education: 78%	NA

Dellar et al., 2021 [23]	Measured	A: etiology B: preventable, preventive measures D: curable, skin, and foot care	Averaged knowledge (i) Knowledge improvement after education (10 ➔ 14 out of 17)	NA

Churko et al. [24]	Measured	A: etiology B: preventive measures, risk factors C: signs and symptoms D: general care	Averaged knowledge (ii) Poor knowledge (score < 75%): 23.1%, good knowledge (score ≥ 75%): 76.9%	Sex, lymphedema education, profession^∗∗∗^, service years^∗∗∗^, health facility location, attitude^∗∗^

Pereira Rios Gerez et al. [25]	Self-reported	A: definition B: preventive measures, at-risk groups C: symptoms, timing of appearance, diagnostic skills D: curable, general care, timing of management, bandaging	Averaged knowledge (i) Self-reported knowledge on definition > symptoms > at-risk population	NA

Bayisenge et al. [26]	Measured	A: etiology B: at-risk groups, preventive measures C: signs and symptoms D: curable	Low knowledge (i) Mean overall knowledge: 58.5% (ii) Knowledge score: CHW (59%) > environmental officers (58%) > physician and nurses/midwives (55%)	Profession, education level, work experience, number of cases treated per month

Abu Sharour [27]	Measured	A: definition, anatomy, pathophysiology B: preventive measures, risk factors, patient education C: assessment and examination D: follow-up appointment	Low knowledge (i) Knowledge score failed (<15): 60%, acceptable (15–20): 25%, good (21–25): 9%, excellent (26–30): 5%	Academic qualification^∗∗∗^, years of experience

Tsuchiya et al. [28]	Self-reported	B: risk factors C: signs and symptoms, early visits D: diet, MLD, weight control, exercise, skin, and wound care	Averaged knowledge (i) Mean overall knowledge: 17.03 (range: 0-26) (ii) Good knowledge on prevention and early signs detection	NA

Mete Civelek et al. [29]	Measured and self-reported	NA	Averaged knowledge (i) Median overall knowledge: 15 (median 25%-75%: 11-18) (ii) Self-reported: very good (1.3%), good (15.3%), middle (64.7%), bad (17.7%), very bad (1.0%)	Gender, years of practice, family physician speciality^∗∗∗^, lymphedema education^∗∗^, very good and good self-reported knowledge^∗^, close relative/friend with BCRL, referral to physical medicine or rehabilitation specialist^∗∗^

Tam et al. [30]	Measured	A: anatomy B: risk factors, preventive measures (familiar with national guideline) C: incidence, symptoms D: curable, exercise, CDT	Averaged knowledge (i) Mean overall knowledge: 9.57 (range: 3-14) (ii) Knowledge score: oncologist (10.66) > surgeon (10.4) > primary care (9.41)	Gender^∗∗^, clinical speciality^∗∗∗^, years of practice, practice care units, physician or nurse, lymphedema education in the past year^∗∗∗^, BCRL referral^∗^

Ryan et al. [31]	Measured and self-reported	A: anatomy, general function, etiology B: risk factors, at-risk groups, patient education C: incidence, symptoms, diagnostic criteria, bioelectrical impendence D: nursing intervention, patient education, deep breathing, exercise, compression therapy, CDT, skin care E: cellulitis	Averaged knowledge (i) Self-reported knowledge on risk reduction > self-management > treatment (ii) Questionnaire knowledge: lowest = general function of lymphatic system (14%), highest = risk reduction and risk factors (88.7%)	Competence in risk reduction and self-management and treatment^∗^, work setting

Yakob et al. [32]	Measured	A: etiology B: preventive measures C: signs and symptoms (general and early)	Averaged knowledge (i) Median overall knowledge: 22 of 39 (low: 54.3% below 22)	Public practice^∗^, favorable attitudes^∗^

Mathews et al. [33]	Self-reported	A: definition (no serious condition), etiology B: preventable, risk factors C: prevalence, symptoms D: curable, general care, refer to proper specialities E: psychosocial difficulties (stigmata, daily activities)	Averaged knowledge (i) Change in 7 of 8 knowledge scores	NA

Morgan et al. [34]	Self-reported and interview	A: edema, etiology B: risk factors C: patient's history, measurement D: general care, MLD, compression therapy, skin care E: AIE, lymphorrhoea, impact on patients	Low knowledge (i) Adequate knowledge: skin care	NA

Rath et al. [35]	Interview	D: curable, home remedies, massage, exercise, bandaging, skin care (wound and foot care) E: acute ADL episodes	Low knowledge (i) Excellent and adequate knowledge: ADL episodes and wound care	NA

Logan et al. [36]	Measured	B: risk factors, preventive measures C: early detection and referral D: curable, limitation of diuretics E: psychosocial difficulties (body image)	Low knowledge (i) Adequate knowledge: limitation of diuretics, body image difficulties, risk factor	Experience^∗∗∗^, profession^∗∗∗^

⸸ A: lymphatic system; B: prevention; C: diagnosis; D: management; E: complications. ^∗^*p* value < 0.05, ^∗∗^*p* value < 0.01, ^∗∗∗^*p* value < 0.001. CHW: community health workers; MLD: manual lymphatic drainage; NA: not available; CDT: complete decongestive therapy; BCRL: breast cancer-related lymphedema; ADL: adenolymphangitis.

**Table 3:** Knowledge gaps, other relevant findings, and suggestions.

Author	Gaps in knowledge	Other relevant findings	Suggestions
Noble-Jones et al. [21]	Assessment and evaluation, exercise, factors affecting therapy, cultural, ethical and legal concerns	(i) Educational needs: compression, surgery, assessment, new advances (ii) Eager for both online and offline educational materials	(i) Supplemental education on genital lymphedema in addition to current training (ii) Better collaboration with other specialities

Omar et al., 2021 [22]	Lymphatic system, general care, lack of knowledge among other HCPs such as physical therapists	(i) Lower experience and competence than knowledge (ii) Low number of certified practitioners (iii) Lack of adequate referral system (iv) 78% eager for professional development opportunities	(i) Self-directed undergraduate educational modules

Dellar et al., 2021 [23]	Etiology	(i) Unfavorable attitudes, high level of stigma, and lack of skills (ii) No significant change in attitude	(i) Improved training (ii) In-service supportive supervision

Churko et al. [24]	Etiology, risk factors	(i) 56% favorable attitudes (ii) 59.7% inadequate knowledge and skills	(i) In-service training

Pereira Rios Gerez et al. [25]	Proper material in lymphedema management, diagnostic skills, preventive measures	(i) Practical knowledge for better practice: lymphatic drainage techniques > therapeutic approach > proper materials > bandaging (ii) Eager for lymphedema education on: therapeutic approach > wounds > treatment > diagnosis > etiology	(i) Education based on highlighted needs and gaps

Bayisenge et al. [26]	Signs and symptoms, at-risk groups	(i) Overall positive attitudes (ii) Gap in practice (wound care and shortage of resources)	(i) Improvement in access to resources (ii) Educational sessions

Abu Sharour [27]	Assessment and examination, follow-up appointment, anatomy, and prevention	(i) 93.3% eager for education in-service	(i) Educational structured

Tsuchiya et al. [28]	Skin care	(i) Good knowledge of referral (ii) 96% eager for education (70% in-service) (iii) Education needs: prevention, LD, and psychosocial care (iv) Low intention to community-based education for cancer survivors	(i) Better understanding of cancer survivors needs

Mete Civelek et al. [29]	NA	(i) Refer BCRL to general surgeon > oncologist > physical medicine > rehabilitation specialist	(i) Undergraduate and postgraduate education (ii) Multidisciplinary approach

Tam et al. [30]	Lower knowledge of primary care providers	(i) Refer BCRL to physical therapist > surgeon > oncologist	(i) Educational interventions especially for PCP

Ryan et al. [31]	General function, risk of BP measurement, deep-breathing exercise impact	(i) Competence as a predictor for practice (ii) Beliefs regarding responsibility of nurses: risk reduction (95%), self-management (68%), and treatment (31%)	(i) Education in nursery school curriculum, conferences, journals, and practice setting (ii) Using National Lymphedema Network and other interest groups (iii) Multidisciplinary approach

Yakob et al. [32]	Etiology, signs and symptoms, stigmata	(i) 100% stigmatizing attitudes (ii) Gap in practice (lack of knowledge and shortage of resources)	(i) Pre- and in-service training (ii) Improvement in access to resources

Mathews et al. [33]	Symptoms, preventable, daily activity difficulties	(i) Positive change in attitude	(i) Capacities of lymphedema management programs (lymphedema roadshow) for education improvement

Morgan et al. [34]	Differential diagnosis (heart failure), lack of clarity of their role, and other professionals	(i) Uncertainty of nurses' role (ii) Lack of adequate skills	(i) Multidisciplinary approach and communication (ii) Educational programs

Rath et al. [35]	Foot care, massage	(i) Gap in practice (foot care)	(i) Peripheral care practitioner orientation

Logan et al. [36]	Early referral, management of lower limb lymphedema, preventive measures	(i) 65.7% eager for education	(i) Appropriate education for specialists

NA: not available.

## Abbreviations

QoL: Quality of life
CVI: Chronic venous insufficiency
BCRL: Breast cancer-related lymphedema
NTDs: Neglected tropical diseases
CDT: Complete decongestive therapy
HCPs: Healthcare practitioners
PRISMA: Preferred Reporting Items for Systematic Reviews and Meta-Analyses
QATSDD: Quality Assessment Tool for Studies with Diverse Designs
MLD: Manual lymphatic drainage.
